# Increased interleukin‐6 levels are associated with atrioventricular conduction delay in severe COVID‐19 patients

**DOI:** 10.1002/joa3.13114

**Published:** 2024-07-31

**Authors:** Riccardo Accioli, Pietro Enea Lazzerini, Viola Salvini, Alessandra Cartocci, Decoroso Verrengia, Tommaso Marzotti, Fabio Salvadori, Stefania Bisogno, Gabriele Cevenini, Michele Voglino, Severino Gallo, Sabrina Pacini, Martina Pazzaglia, Angelica Tansini, Ambra Otranto, Franco Laghi‐Pasini, Maurizio Acampa, Mohamed Boutjdir, Pier Leopoldo Capecchi

**Affiliations:** ^1^ Department of Medical Sciences, Surgery and Neurosciences University of Siena Siena Italy; ^2^ Division of Internal Medicine and Geriatrics, Electroimmununology Unit University Hospital of Siena Siena Italy; ^3^ Department of Medical Biotechnologies University of Siena Siena Italy; ^4^ Stroke Unit University Hospital of Siena Siena Italy; ^5^ VA New York Harbor Healthcare System SUNY Downstate Health Sciences University New York New York USA; ^6^ NYU Grossman School of Medicine New York New York USA

**Keywords:** atrioventricular block, COVID‐19, interleukin‐6, PR‐interval, PR‐segment

## Abstract

**Background:**

Severely ill patients with coronavirus disease 2019 (COVID‐19) show an increased risk of new‐onset atrioventricular blocks (AVBs), associated with high rates of short‐term mortality. Recent data suggest that the uncontrolled inflammatory activation observed in these patients, specifically interleukin (IL)‐6 elevation, may play an important pathogenic role by directly affecting cardiac electrophysiology. The aim of our study was to assess the acute impact of IL‐6 changes on electrocardiographic indices of atrioventricular conduction in severe COVID‐19.

**Methods:**

We investigated (1) the behavior of PR‐interval and PR‐segment in patients with severe COVID‐19 during active phase and recovery, and (2) their association with circulating IL‐6 levels over time.

**Results:**

During active disease, COVID‐19 patients showed a significant increase of PR‐interval and PR‐segment. Such atrioventricular delay was transient as these parameters rapidly normalized during recovery. PR‐indices significantly correlated with circulating IL‐6 levels over time. All these changes and correlations persisted also in the absence of laboratory signs of cardiac strain/injury or concomitant treatment with PR‐prolonging drugs, repurposed or not.

**Conclusions:**

Our study provides evidence that in patients with severe COVID‐19 and high‐grade systemic inflammation, IL‐6 elevation is associated with a significant delay of atrioventricular conduction, independent of concomitant confounding factors. While transient, such alterations may enhance the risk of severe AVB and associated short‐term mortality. Our data provide further support to current anti‐inflammatory strategies for severe COVID‐19, including IL‐6 antagonists.

## Graphical abstract

In patients with severe coronavirus disease 2019 and high‐grade systemic inflammation, interleukin‐6 elevation is associated with a significant delay of atrioventricular conduction, independent of concomitant confounding factors. While transient, such alterations may enhance the risk of severe atrioventricular block and associated short‐term mortality.

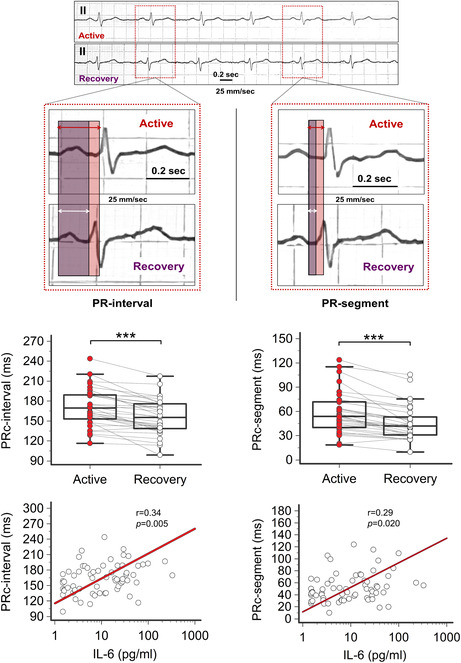

## 1 INTRODUCTION

Cardiac involvement deeply impacts the prognosis of coronavirus disease 2019 (COVID‐19), accounting for up to 30% of the complications observed in severe forms.^1^, ^2^ A particularly relevant role seems to be played by cardiac rhythm disturbances, including atrial fibrillation, ventricular arrhythmias, and bradyarrhythmias,^3^ overall occurring in ~10%–20% of patients during the acute phase of the disease.^4^, ^5^ Specifically, it has been reported that new‐onset atrioventricular blocks (AVBs) develop in approximately 5% of severely ill patients, including second (II°)‐ and third (III°)‐degree AVB (1%–2%), and associate with high rates of short‐term mortality.^6^, ^7^, ^8^, ^9^ While the underlying mechanisms are poorly understood, several pathogenic factors are postulated, including acute myocardial injury/strain, indirectly induced by pneumonia‐dependent hypoxia or directly by virus entry in cardiac cells, and toxicity of repurposed “off‐label” medications, such as antimalarials, remdesivir, protease inhibitors, and azithromycin.^10^, ^11^, ^12^, ^13^, ^14^, ^15^ However, it is increasingly recognized that in a number of cases of COVID‐19‐associated AVB, the conduction defect occurs even in the absence of any of the aforementioned factors.^16^, ^17^, ^18^, ^19^

The PR‐interval measured on the surface electrocardiogram (ECG) is the most commonly used parameter to evaluate atrioventricular (AV) conduction in the clinical practice, as it represents a well‐accepted surrogate of the physiological delay between atrial and ventricular activation. Specifically, a prolongation of PR‐interval >200 ms is defined as first‐degree (I°) AVB, in the recent years increasingly recognized to be an independent risk factor in the general population for severe AVBs requiring pacemaker implantation, but also for other cardiac events such as heart failure, atrial fibrillation, ventricular arrhythmias, and cardiac death.^20^, ^21^, ^22^, ^23^ Some studies preliminarily reported that PR‐interval is prolonged in COVID‐19 patients^9^, ^18^ and associated with increased mortality.^9^

Electrophysiological mechanisms underlying AV conduction are very complex and critically dependent on the function of a wide number of ion channels, including L‐type calcium channels and connexin‐formed gap junctions.^24^ In this regard, Hulsmans et al.^25^ recently provided strong evidence that in physiological conditions, AV conduction is crucially modulated by inflammation‐related cells, that is, cardiac macrophages, located in the distal part of the AV node and electrically coupled with conduction cardiomyocytes via gap junctions containing connexin‐43.

Inflammatory activation is increasingly recognized as an important pathogenic factor for cardiac arrhythmias via multiple arrhythmogenic effects of inflammatory cytokines, including both direct activities on cardiac electrophysiology and indirect systemic effects.^26^, ^27^, ^28^ Specifically, accumulating evidence indicates that these molecules, primarily tumor necrosis factor‐alpha, interleukin (IL)‐1, and IL‐6, can promote conduction disturbances development by inducing gap‐junction dysfunction (hour to days) and cardiac fibrosis (weeks to months), in turn slowing the impulse propagation throughout the working and conducting myocardium.^28^ Accordingly, a recent translational study involving patients with acute non‐COVID‐19 infections, mostly bacterial pneumonia, and other inflammatory diseases provided evidence for an association between AV conduction times, IL‐6 levels, and cardiac connexin‐43 expression.^29^ Moreover, several cases of acute influenza infection complicated with severe AV block have been reported, in most cases spontaneously reversing when disease and inflammatory activation recovered.^30^, ^31^, ^32^, ^33^, ^34^, ^35^

Based on this background, and in consideration of the massive systemic release of cytokines, particularly IL‐6, characterizing most cases of severe COVID‐19,^36^, ^37^, ^38^ we hypothesized that in these patients, inflammatory activation, specifically IL‐6 elevation, can exert a significant independent role in acutely delaying AV conduction. Thus, the aim of our study was to assess: (i) the changes of PR‐interval and other ECG indices of AV conduction in patients with severe COVID‐19 during active phase and recovery, and (ii) their association with circulating IL‐6 levels over the time.

## 2 MATERIALS AND METHODS

The local ethical committee (Comitato Etico Regionale per la Sperimentazione Clinica della Regione Toscana, Sezione Area Vasta Sud Est) approved the research, and patients from all cohorts gave their oral and written informed consent according to the principles of the Declaration of Helsinki.

### 2.1 Study populations

Thirty‐three patients with severe COVID‐19 admitted to our University Hospital were prospectively collected. Diagnosis was based on the presence of: (1) symptoms indicative of COVID‐19, and (2) positive nasopharyngeal swab by polymerase chain reaction assays for severe acute respiratory syndrome coronavirus (SARS‐CoV‐2).^39^ Occurrence of respiratory failure at any time during hospitalization requiring oxygen‐therapy or mechanical ventilation or need for intensive care unit (ICU) treatment was additionally required to specifically fulfil the diagnosis of severe disease.^40^ A blood withdrawal and an ECG recording were simultaneously performed in all patients during both active disease and recovery. Specifically, we defined the recovery phase as reached, when therapeutic interventions resulted in clinical improvement associated with significant IL‐6 levels reduction, >60% when compared to baseline. Patients who were under treatment with medications potentially affecting AV conduction,^13^, ^29^ including antiarrhythmics (class I or III antiarrhythmics, beta‐blockers, nondihydropyridine calcium channel blockers), phenytoin, lithium, or COVID‐19 repurposed drugs (i.e., remdesivir, chloroquine, hydroxychloroquine, lopinavir/ritonavir, and azithromycin), were excluded only if they assumed these drugs during the active disease, but not in the recovery phase (while keeping patients continuing the same medication throughout). The demographic and clinical features of the COVID‐19 cohort are detailed in Table 1.

**TABLE 1:** Demographic and clinical characteristics of COVID‐19 patients and controls.

	Patients	Controls
Patients, *n*	33	18
Age, years	63.5 ± 12.3	63.5 ± 11.5
Females, *n*	11 (33%)	6 (33%)
Clinical features		
Dyspnea	15/33 (46%)	0/18
Respiratory failure	11/33 (33%)	0/18
Fever	10/33 (30%)	0/18
Gastrointestinal symptoms	5/33 (15%)	0/18
Cough/sneeze	5/33 (15%)	0/18
Osteoarticular symptoms	2/33 (6%)	0/18
Asthenia	2/33 (6%)	0/18
Lipothymia	1/33 (3%)	0/18
Severe COVID‐19, *n*	33/33 (100%)	0/18
Respiratory support	33/33 (100%)	0/18
ICU admission	3/33 (9%)	0/18
Death	1/33 (3%)	0/18
Respiratory support, *n*	33/33 (100%)	0/18
*Oxygen therapy*	33/33 (100%)	0/18
Nasal cannulas	33/33 (100%)	0/18
VentiMask	31/33 (94%)	0/18
HFNC	24/33 (23%)	0/18
*Mechanical support*	20/33 (61%)	0/18
CPAP	20/20 (100%)	0/18
OTI	2/20 (1%)	0/18
Comorbidities, *n*	16/33 (48%)	0/18
Cardiovascular disease	14/16 (88%)	0/18
Hypertension/LVH	13/14 (93%)	0/18
CAD	2/14 (14%)	0/18
Obesity	3/33 (9%)	0/18
COPD	2/33 (6%)	0/18
Electrolyte imbalances^a^	0/33	0/18
PR‐prolonging drugs	10/33 (30%)	0/18
Repurposed COVID‐19 drugs	7/10 (70%)	0/18
Azithromycin	4/7 (57%)	0/18
Remdesivir	3/7 (43%)	0/18
Classic PR‐prolonging drugs	4/10 (40%)	0/18
Beta‐blockers	4/4 (100%)	0/18
Amiodarone	1/4 (25%)	0/18

*Note*: Values are expressed as mean ± standard deviation or frequency count and percentages.

Abbreviations: CAD, coronary artery disease; COPD, chronic obstructive pulmonary disease; COVID‐19, coronavirus disease 2019; CPAP, continuous positive airway pressure; HFNC, high‐flow nasal cannula; ICU, intensive care unit; LVH, left ventricular hypertrophy; OTI, orotracheal intubation.

^a^ Based on the following reference values: sodium (135–145 mEq/L), potassium (3.5–5.5 mEq/L), calcium (8–11 mg/dL), and magnesium (1.5–2.5 mg/dL).

An additional sample constituted by 18 healthy controls (i.e., without comorbidities, electrolyte imbalances and PR‐prolonging drugs) comparable with COVID‐19 patients in terms of age and gender (*p* ≥ .99 in both cases, unpaired *t*‐test or the Fisher's exact test; Table 1) was collected as a confirmatory group for ECG and laboratory parameters. All these subjects underwent a blood withdrawal and an ECG recording, simultaneously performed at the time of enrollment (Table 1).

### 2.2 ECG recordings

PR‐interval, representing the sum of P‐wave duration and PR‐segment, is universally used to quantify the duration of AV conduction. Specifically, P‐wave duration mirrors the propagation of electric impulses through atria, while PR‐segment represents the period of time in which the same impulses are slowed in the AV node, before entering the bundle branches. Hence, measurement of PR‐segment constitutes a more precise tool to discretely assess AV nodal conduction. In all subjects, heart rate (HR), RR‐interval, PR‐interval, and PR‐segment were manually measured on a standard 12‐lead ECG (25 mm/s and 10 mV/cm; sampling rate 1 kHz; Cardioline ECT WS 2000, Remco Italia, Vignate‐Milano, Italy), in supine position and during spontaneous breathing. ECG tracings were scanned and digitized to reach greater precision in detecting and measuring PR‐interval and PR‐segment. All ECG parameters were measured from 3 nonconsecutive beats (mean value).

PR‐interval duration was measured from the onset of P‐wave deflection to the onset of QRS complex. It is well recognized that PR‐interval duration is modulated by HR changes (inverse relationship), accounting for the significant beat‐to‐beat or day‐to‐day variability observed in physiological conditions. To improve the intra‐subject stability of this parameter over the time, PR‐interval was then corrected for HR by means of Soliman‐Rautaharju's formula (PR‐interval + 0.26 (HR – 70), in subjects <60 years; PR‐interval + 0.42 (HR – 70) in subjects >60 years) to obtain the heart‐corrected PR‐interval (PRc‐interval).^41^ According to the current American College of Cardiology Foundation/American Heart Association/Heart Rhythm Society guidelines, a PR‐interval >200 ms was considered abnormal and defined as first‐degree AV block (I°AVB).^42^ Additionally, a PR‐interval >99% confidence interval [CI] observed in the general population when stratified for age and gender^43^ was considered abnormal.

PR‐segment duration was calculated from the end of P‐wave deflection to the onset of QRS complex. Although no formulas adjusting PR‐segment for HR are currently available, the evidence that P‐wave duration is not significantly affected by HR^44^ implies that PR‐segment is the specific sub‐component of PR‐interval which actually requires HR‐adjustment. Hence, Soliman‐Rautaharju's formula^41^ was also applied to PR‐segment to estimate, for research purposes, heart‐corrected PR‐segment (PRc‐segment).

Finally, since measurements of the PR‐intervals and PR‐segments could be subject to inter‐ and intra‐observer variability, we evaluated intra‐rater and inter‐rater reliability by estimating the intraclass correlation coefficient (ICC) and its 95% confidence interval based on the initial 20 measurements of PR‐intervals and PR‐segments conducted by two investigators (M.A. and R.A.). Given that the assessment of PR‐intervals and PR‐segments showed robust repeatability and reproducibility within our study cohort, the following measurements were performed by a single investigator (M.A.) who was blinded to the clinical status of the investigated subject. A detailed description of ICC estimation is provided as Supplemental Information (Supplemental Methods—Data S1).

### 2.3 Laboratory analysis

Blood samples were centrifuged at 3000xg, and sera were stored at −80°C.

IL‐6, C‐reactive protein (CRP), NT‐pro‐brain natriuretic peptide (BNP), and troponin were measured by an electrochemiluminescence‐based immunoassay (COBAS‐8000 platform, Roche Diagnostics GmbH; Mannheim, Germany), and values were reported as mg/dl (CRP, reference values <0.5), ng/mL (troponin, r.v. <15), or pg/mL (IL‐6, r.v. <7.1; BNP, r.v. <500), respectively.

### 2.4 Statistical analysis

For the active vs recovery phase analyses, which represents the primary outcome, a sample size of 33 patients was estimated based on the two‐sided Wilcoxon test, and considering a first type error of 0.05, a power of 0.80, and a medium effect size = 0.5. Moreover, a cohort of 18 controls was selected based on a two‐sided Mann–Whitney test to obtain a confirmatory group of healthy patients, with first type error of 0.05, power of 0.80, a large effect size of 0.85, and a 2:1 ratio between cases and controls. Sample size calculation was estimated with G*Power. Parametric or nonparametric studies were carried out based on the Kolmogorov–Smirnov test. To compare active and recovery phase, the paired *t*‐test or the Wilcoxon test was used; comparisons between patients and controls were performed with the unpaired *t*‐test or the Mann–Whitney test. The Fisher's exact test or the McNemar test was used for qualitative variables. Correlation analyses were performed with Spearman's rank test. A *p*‐value ≤ .05 was considered to be significant (GraphPad InStat). The Bonferroni's correction for multiple tests was applied when COVID‐19 patients during active disease and recovery were compared to controls.

## 3 RESULTS

### 3.1 COVID‐19 patients' characteristics

According to the inclusion criteria, 33 patients (mean age 62.9 ± 12.5; 70% males) with severe COVID‐19 were consecutively collected during the third wave of COVID‐19 pandemic. Most common symptoms on admission were dyspnea (50%), respiratory failure (33%), and fever (27%). During hospitalization, all patients needed oxygen therapy including mechanical ventilation in ~2/3 of cases. Three subjects required ICU admission, and one of them eventually died. Significant comorbidities were found in half of the patients, more commonly cardiovascular disease (88%), while 10 subjects (30%) were concomitantly treated with drugs potentially delaying AV conduction (Table 1).

### 3.2 PR‐interval in patients with severe COVID‐19 and correlation with inflammatory markers

**FIGURE 1:**
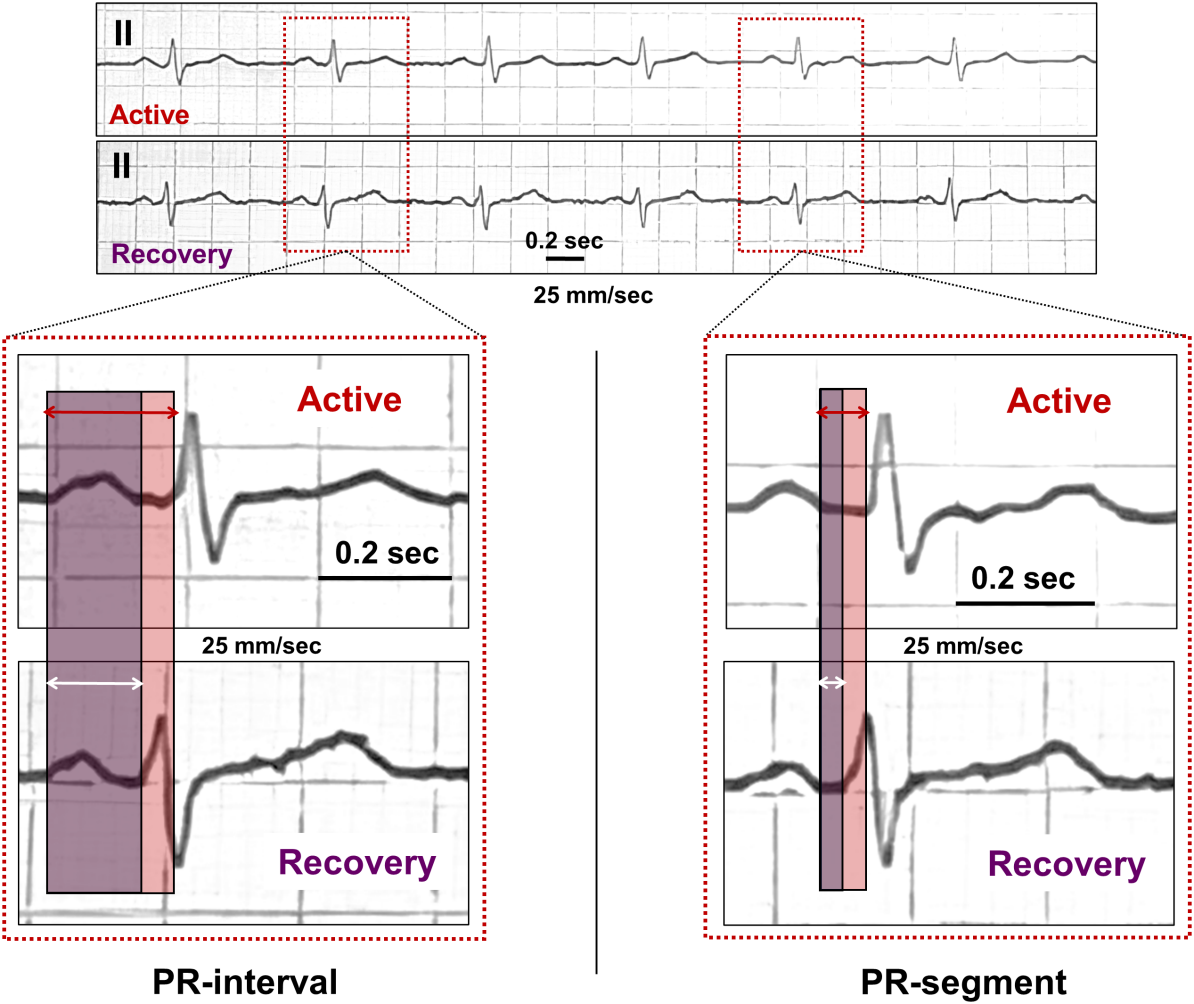
Representative ECG strips (lead II) of a 57‐year‐old COVID‐19 patient, during active disease (PRc‐interval: 170.3 ms; PRc‐segment: 43.6 ms; IL‐6: 48.4 ng/mL) and after a 7‐day treatment with 80 mg methylprednisolone (PRc‐interval: 134.7 ms; PRc‐segment: 33.1 ms; IL‐6: 9.2 ng/mL), Colored areas in light red and light blue (and associated arrows in red and withe) indicate PRc‐interval and PRc‐segment in active and recovery phase, respectively.

**FIGURE 2:**
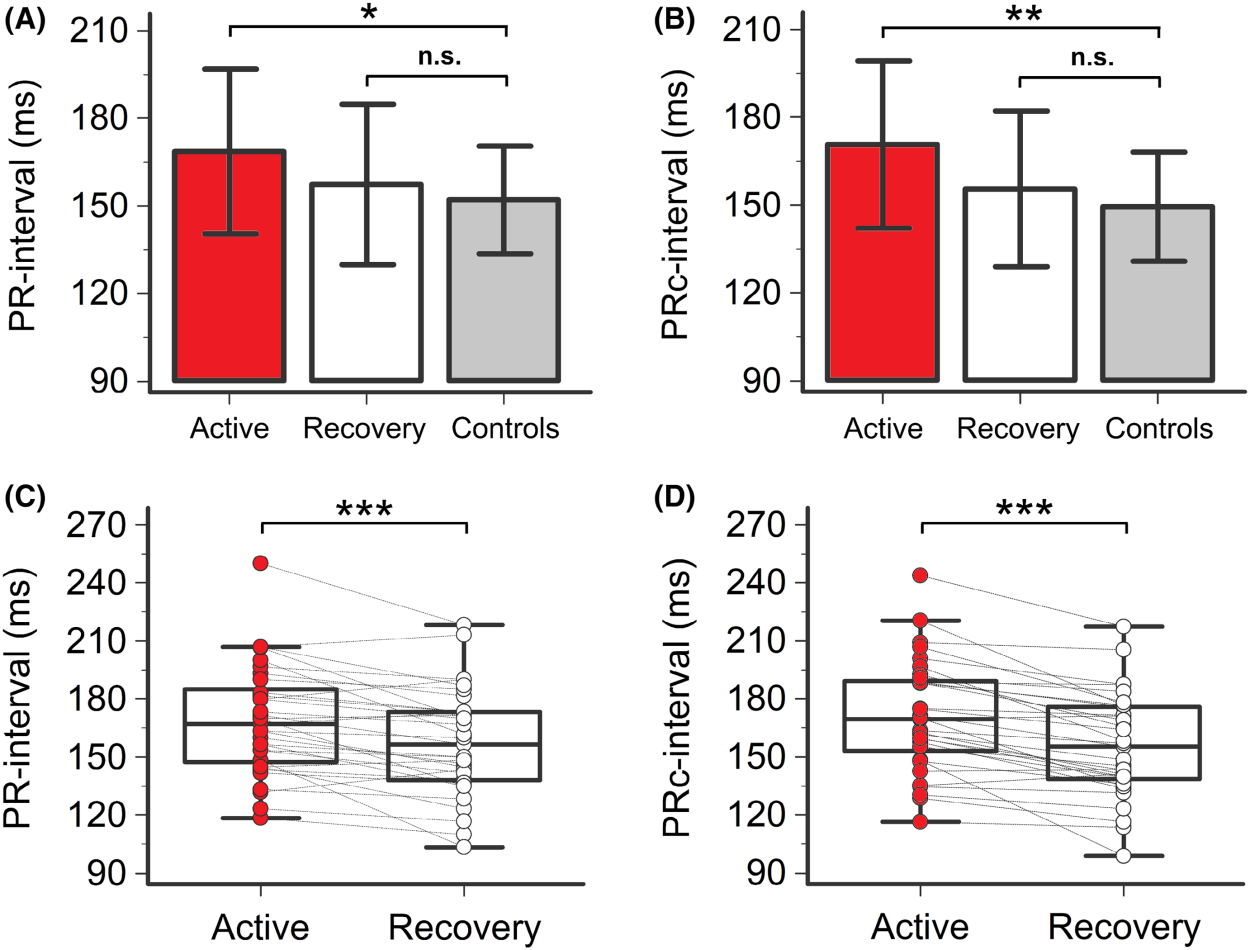
PR‐interval and PRc‐interval in patients with COVID‐19, during active disease and recovery, and controls. (A) Comparison of PR‐interval in patients with COVID‐19, during active disease and recovery, and controls; two‐tailed unpaired *t*‐test, *p < .05, n.s. *p* > .05. (B) Comparison of PRc‐interval in patients with COVID‐19, during active disease and recovery, and controls; two‐tailed unpaired *t*‐test, **p < .025, n.s. *p* > .05. (C) PR‐interval in patients with COVID‐19, during active disease and recovery; two‐tailed paired *t*‐test, ***p < .001. (D) PRc‐interval in patients with COVID‐19, during active disease and recovery; two‐tailed paired *t*‐test, ***p < .001. Patients, *n* = 33; controls, *n* = 18.

During the active phase, patients with severe COVID‐19 presented with a mean PR‐interval duration of 168 ms, with a ~ 16 ms difference when compared to controls (168.6 ± 28.3 vs. 152.0 ± 18.4 ms, *p* = .029) (Table 2; Figures 1 and 2A; Table S1). I°AVB was found in five COVID‐19 patients (5/33 [15%], in one subject severe prolongation >240 ms), but in none of controls. Moreover, 39% of COVID‐19 patients showed PR‐interval values >99% CI for age and gender in the general population,^43^ a percentage almost twice when compared to controls (22%) (Table 2; Table S1). Importantly, patients with active COVID‐19 also showed a higher HR than controls (71.2 ± 13.0 vs. 62.8 ± 9.0 ms; *p* < .001, two‐tailed unpaired *t*‐test), a change most likely driven by inflammation‐induced sympathetic activation.^45^, ^46^ In order to exclude that this factor might have underestimated the differences (PR‐interval and HR have an inverse relationship),^41^ PR‐interval was then corrected for the HR using Soliman‐Rautaharju's formula^41^ to obtain PRc‐interval. Accordingly, correction underscored differences by showing that PRc‐interval mean values were significantly increased in active COVID‐19 patients compared to controls (170.7 ± 28.6 vs. 149.5 ± 18.6 ms, +21.2 ms; *p* = .007) (Table 2; Figure 2B).

**TABLE 2:** Changes in laboratory and electrocardiographic parameters in patients with COVID‐19 (*n* = 33), during active disease and after therapeutic interventions resulting in an IL‐6 decrease >60% when compared to the baseline.

	Active	Recovery	*p*
CRP, mg/dL (r.v. <0.5)	9.9 ± 8.2	0.9 ± 1.0	**<.001**
IL‐6, pg/mL (r.v. <7.1 pg/mL)	46.7 ± 64.8	3.8 ± 28.2	**<.001**
Heart rate, bpm	76.1 ± 13.0	63.6 ± 10.3	**<.001**
RR, ms	809.0 ± 142.4	956.9 ± 148.9	**<.001**
PR‐interval, ms	168.6 ± 28.3	157.4 ± 27.4	**<.001**
PRc‐interval, ms	170.7 ± 28.6	155.5 ± 26.6	**<.001**
PR‐segment, ms	55.9 ± 25.5	47.8 ± 21.7	**<.001**
PRc‐segment, ms	58.0 ± 25.8	45.9 ± 20.9	**<.001**
P‐wave duration, ms	112.2 ± 14.3	109.6 ± 13.9	.23
Patients with PR‐interval >99% CI^a^, *n*	13 (39%)	8 (24%)	.13
Patients with PRc‐interval >99% CI^a^, *n*	15 (45%)	8 (24%)	**.020**
Patients with I°AVB^b^, *n*	5 (15%)	2 (6%)	.15
Troponin, ng/mL (r.v. <30)	16.2 ± 30.1	11.5 ± 11.7	.55
Patients with increased troponin, *n*	3 (9%)	2 (6%)	1.0
BNP, pg/mL (r.v. <500)	474.8 ± 554.6	305.4 ± 317.8	.35
Patients with increased BNP, *n*	8 (24%)	7 (21%)	1.0
paO_2_, mmHg (r.v.70–100)	90.8 ± 39.9	97.9 ± 32.0	.10
paCO_2_ mmHg (r.v. 35–45)	35.6 ± 3.8	36.4 ± 4.9	.46
pH (r.v.7.35–7.45)	7.46 ± 0.0	7.45 ± 0.0	.52
P/F (r.v. >4.0)	2.1 ± 1.0	2.7 ± 0.8	**.027**

*Note*: Values are expressed as mean ± standard deviation or frequency count and percentages. Differences were evaluated by the two‐tailed Student's paired “*t*”‐test, the two‐tailed Wilcoxon matched‐pairs test, the unpaired *t*‐test, the Mann–Witney *t*‐test, or the McNemar test. Statistically significant values (*p* < .05) are reported in bold.

Abbreviations: BNP, NT‐pro‐brain natriuretic peptide; CRP, C‐reactive protein; IL‐6, interleukin‐6; P/F, paO_2_/FiO_2_ ratio; PRc‐interval, corrected PR‐interval based on the Soliman's formula; PRc‐segment, corrected PR‐segment based on the Soliman's formula; r.v., reference values; RR, RR interval.

^a^ PR‐interval and PRc‐interval >99% confidence interval (CI) for age and gender in the general population.

^b^ PR‐interval and/or PRc‐interval >200 ms.

**FIGURE 3:**
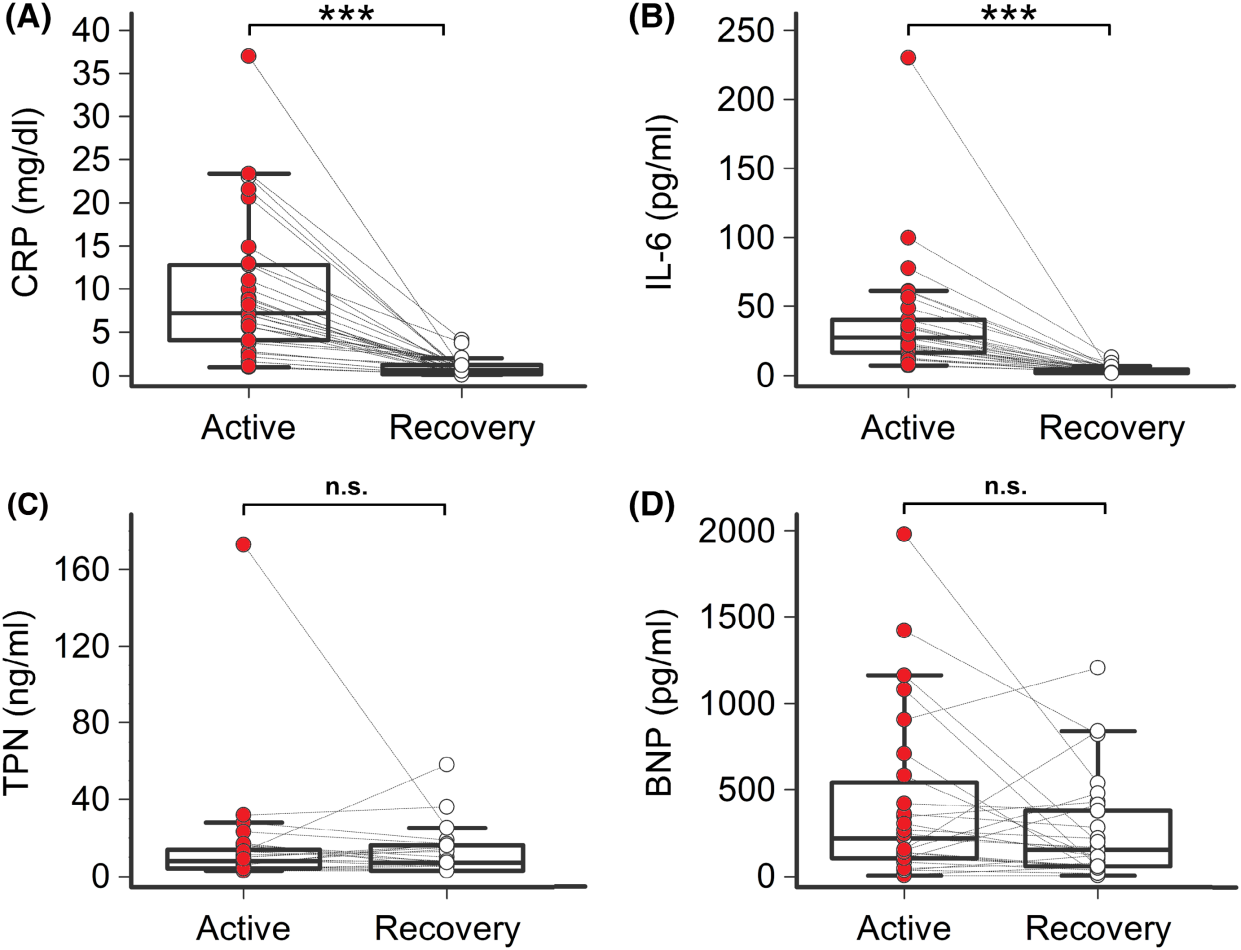
C‐reactive protein (CRP), interleukin‐6 (IL‐6), troponin (TPN), and NT‐pro‐brain natriuretic peptide (BNP) in patients with COVID‐19, during active disease and recovery. (A) CRP; Wilcoxon *t*‐test, ***p < .001. (B) IL‐6; Wilcoxon *t*‐test, ***p < .001. (C) TPN, Mann–Whitney, n.s. *p* > .05. (D) BNP; Mann–Whitney, n.s. *p* > .05. Patients, *n* = 33.

**FIGURE 4:**
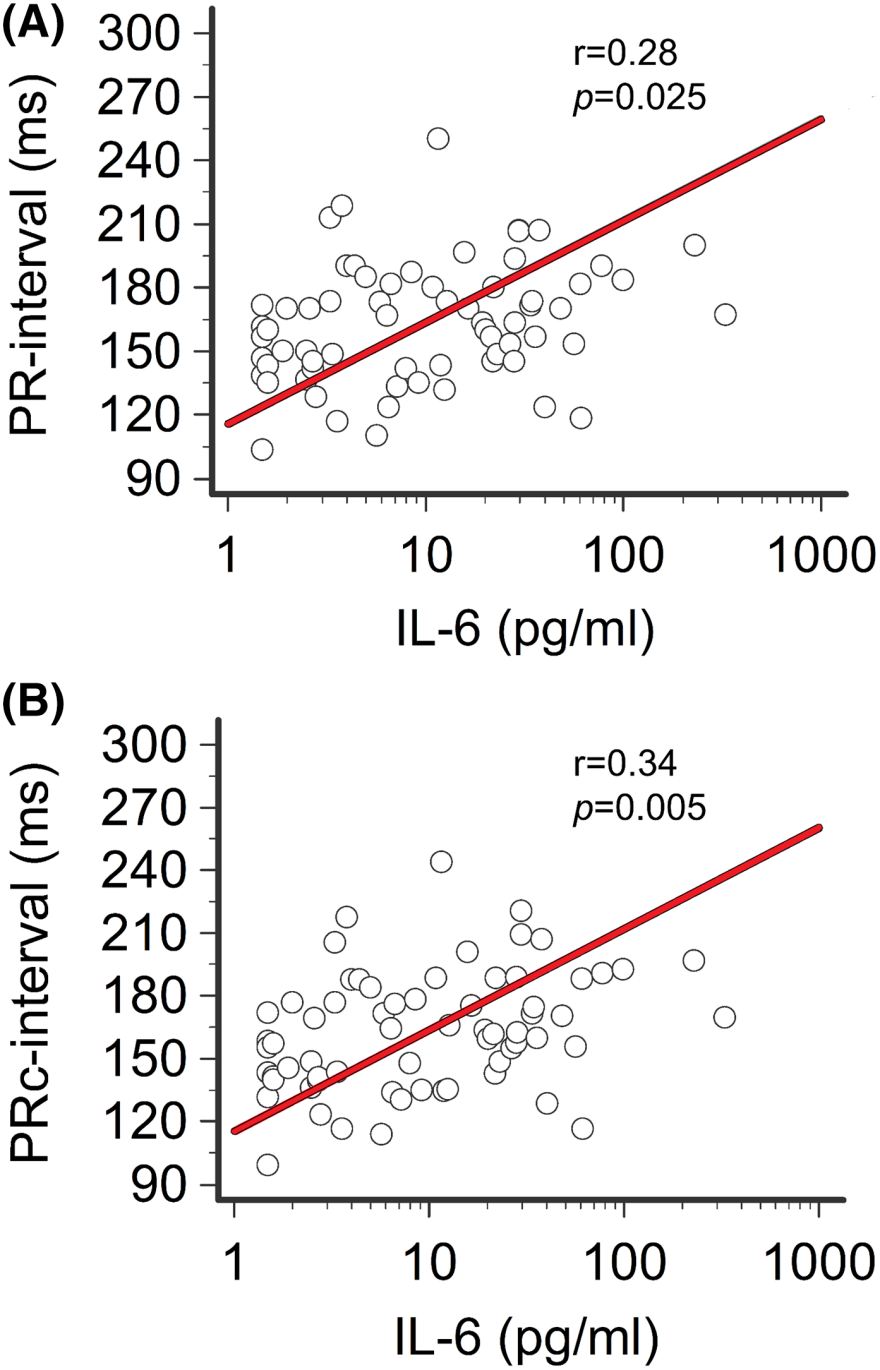
Correlation between PR‐interval/PRc‐interval and IL‐6 in patients with COVID‐19 over time. (A) Relationship between PR‐interval and IL‐6 levels. (B) Relationship between PRc‐interval and IL‐6 levels. Spearman's rank correlation. Patients, *n* = 33.

During hospitalization, all patients received anti‐inflammatory treatment with medium–high doses of glucocorticoids (methylprednisolone or dexamethasone; median starting daily dose: 80 and 8 mg, respectively); four subjects were additionally treated with an immunosuppressive drug, that is, the Janus‐kinase inhibitor baricitinib. Seven patients only were treated with repurposed anti‐COVID‐19 medications (azithromycin, *n* = 4, remdesivir, *n* = 3; no patients received antimalarials or protease inhibitors). Treatment resulted in a rapid (mean time 10.6 ± 8.4 days, median 7 days) and evident decrease in both CRP (mean reduction 87%) and IL‐6 (mean reduction 86%) levels (Figure 3; Table 2). As anticipated (hepatic CRP synthesis is mostly IL‐6 driven), CRP and IL‐6 strongly correlated over time (*r* = .75, *p* < .001, Spearman's rank test). In this recovery phase, a significant reduction in PR‐interval (mean ΔPR‐interval = −11.3 ms, *p* < .001) and PRc‐interval (mean ΔPRc‐interval = −15.2 ms, *p* < .001) was concomitantly observed, until reaching values comparable to controls. Accordingly, the frequency of I°AVB was reduced (2/33 [6%]), with ~2/3 of the patients who presented with I°AVB during active disease showing a normal AV conduction. Moreover, the percentage of patients showing PRc‐interval values >99% CI for age and gender significantly decreased to 24% (*p* = .02), a frequency not different from that observed in controls (*p* = .73, Fisher's exact test) (Figure 2A–D; Table 2; Table S1). Circulating IL‐6 significantly correlated with PR‐interval (*r* = .29, *p* = .025) and PRc‐interval (*r* = .34, *p* = .005) values over time (Figure 4). Conversely, no significant association with CRP levels was found (Table S6). All the other laboratory parameters, including cardiac strain/injury indices (troponin, BNP), blood gases, and pH mean values, remained stable within the reference range throughout the entire study period (Figure 3; Table 2).

Some patients (13/33, 38%) showed abnormal BNP and/or troponin levels in active and/or recovery phase, although these changes did not reach statistical significance. Moreover, ~1/3 (10/30, 33%) of the subjects were concomitantly treated with COVID‐19 repurposed or classic PR‐prolonging drugs, although stably throughout the study time. To rule out that these factors, by negatively affecting AV conduction, may have in some way biased the results, multiple sensitivity analyses were performed by selectively evaluating patients without signs of cardiac injury/strain, without COVID‐19 repurposed medications, without classic PR‐prolonging drugs, and without any of the above PR‐prolonging risk factors, respectively. Consistently with what observed in the whole COVID‐19 population, in all cases PR/PRc‐interval rapidly and significantly reduced in the recovery phase (Tables S2–S5; Figure S1). Moreover, a significant correlation between PR/PRc‐interval and IL‐6 levels was persistently found, with *r* values even stronger than those observed in the entire cohort. In particular, when patients without concomitant risk factors were selectively considered, the strength of the correlation significantly increased up to *r* = .38 for PRc‐interval (Table 3). A similar trend was also observed for CRP levels, whose correlation with PR/PRc‐interval in this subset of patients approached statistical significance (PR‐interval: *r* = .32, *p* = .083; PRc‐interval: *r* = .34, *p* = .069; Table S6). Nonetheless, patients with concomitant PR‐prolonging risk factors tended to have higher mean PR/PRc‐interval values in both active and recovery phases when compared to those without, particularly in the presence of elevated levels of BNP/troponin (Figure S1).

**TABLE 3:** Correlations between PR‐indices and IL‐6 in the overall COVID‐19 population and in COVID‐19 patients without specific or any PR‐prolonging risk factor.

	PR‐interval	PRc‐interval	PR‐segment	PRc‐segment
All patients (*n* = 33)	*r* = .28	*r* = .34	*r* = .21	*r* = .29
	***p* = .025**	***p* = .005**	*p* = .085	***p* = .020**
Patients without repurposed COVID‐19 drugs (*n* = 26)	*r* = .25	*r* = .31	*r* = .24	*r* = .30
	*p* = .074	***p* = .024**	*p* = .086	***p* = .027**
Patients without classic PR‐prolonging drugs (*n* = 29)	*r* = .30	*r* = .35	*r* = .20	*r* = .27
	***p* = .020**	***p* = .007**	*p* = .14	*p* = **.042**
Patients without cardiac strain/injury^a^ (*n* = 20)	*r* = .35	*r* = .39	*r* = .26	*r* = .33
	***p* = .026**	***p* = .013**	*p* = .10	***p* = .035**
Patients without any PR‐prolonging risk factor (*n* = 15)	*r* = .34	*r* = .38	*r* = .36	*r* = .40
	*p* = .064	***p* = .041**	*p* = .053	***p* = .030**

*Note*: Correlations were evaluated by the Spearman's rank correlation. Statistically significant values (*p* < .05) are reported in bold.

Abbreviations: BNP, NT‐pro‐brain natriuretic peptide; IL‐6, interleukin‐6; P/F, paO_2_/FiO_2_ ratio; PRc‐interval, corrected PR‐interval based on the Soliman's formula; PRc‐segment, corrected PR‐segment based on the Soliman's formula; r.v., reference values; RR, RR interval.

^a^ Patients with both normal troponin (<30 ng/mL) and BNP (<500 pg/mL) levels.

### 3.3 PR‐segment in patients with severe COVID‐19 and its relationship with inflammatory markers

The PR‐interval reflects the cumulative time requested for both intra‐atrial and AV electrical impulse propagation. In consideration of previous data which demonstrated that IL‐6 can significantly affect intra‐atrial conduction,^47^ PR‐segment was also evaluated, to better quantify the specific impact of inflammation on AV conduction.

**FIGURE 5:**
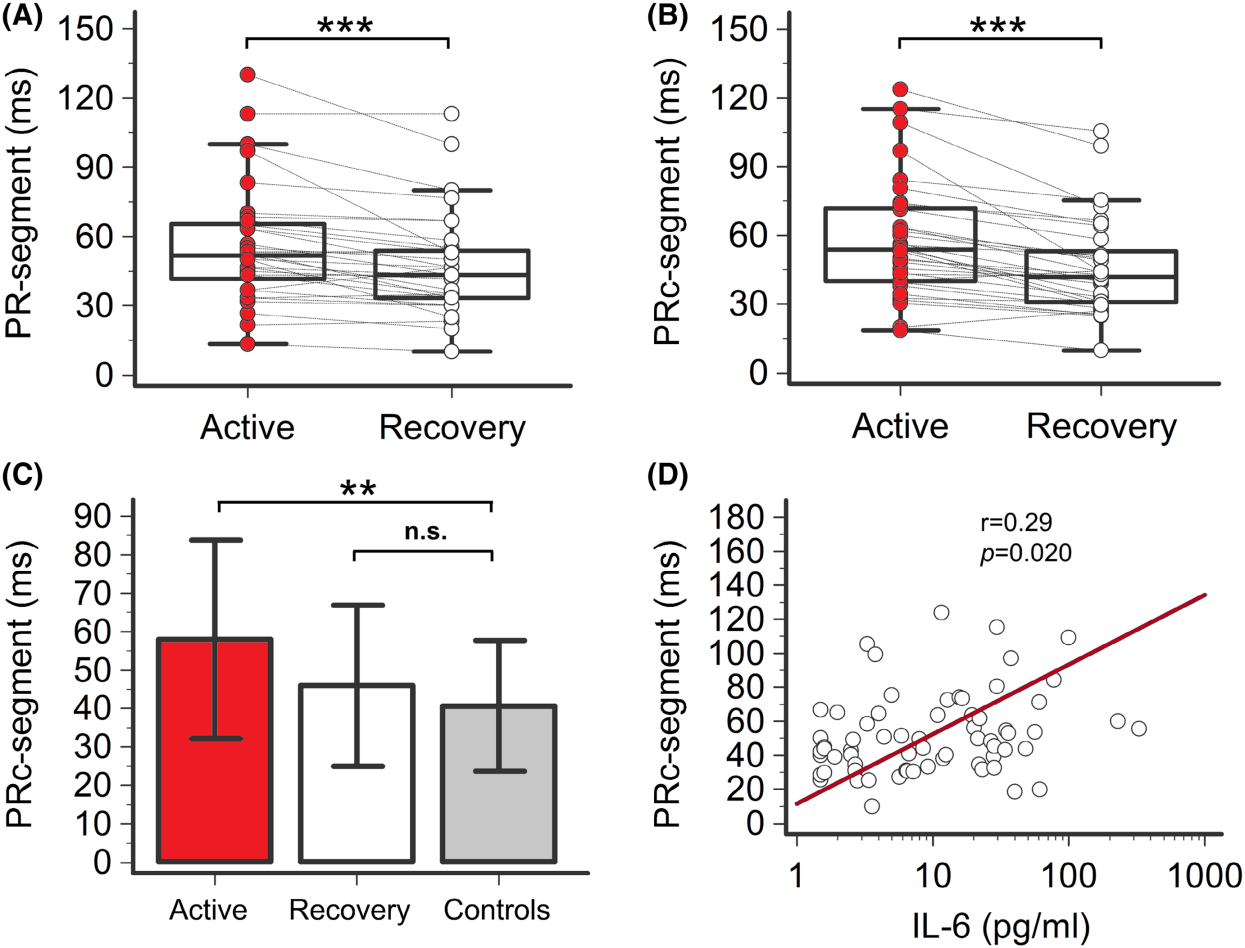
PR‐segment and PRc‐segment in patients with COVID‐19, during active disease and recovery, and controls. (A) PR‐segment in patients with COVID‐19, during active disease and recovery; two‐tailed paired *t*‐test, ***p < .001. (B) PRc‐segment in patients with COVID‐19, during active disease and recovery; two‐tailed paired *t*‐test, ***p < .001. (C) Comparison of PRc‐segment in patients with COVID‐19, during active disease and recovery, and controls; two‐tailed unpaired *t*‐test, **p < .025, n.s. *p* > .05. (D) Relationship between PRc‐segment and IL‐6 levels. Spearman's rank correlation. Patients, *n* = 33.

In the active phase, COVID‐19 patients showed a mean PR‐segment duration of 56 ms, with a ~ 12 ms change when compared to controls (55.9 ± 25.5 vs. 43.7 ± 17.2 ms, *p* = .042), a difference which further increased after HR correction by using Soliman‐Rautaharju's formula (PRc‐segment: 58.0 ± 25.8 vs. 40.7 ± 17.0 ms; mean ΔPRc‐segment =17.3 ms; *p* = .0097, two‐tailed Mann–Whitney test) (Table 2; Figures 1 and 5A–C; Table S1). After treatment, both PR‐segment and PRc‐segment were significantly reduced (ΔPR‐segment = −8.1 ms, from 55.9 to 47.8 ms, *p* < .001; ΔPRc‐segment = −12.1 ms, from 58.0 to 45.9 ms, *p* < .001) finally overlapping values observed in controls (Table 2; Figure 5A,B; Table S1). Again, all these changes were consistently observed throughout the several sensitivity analyses performed (Tables S2–S5; Figure S2). Moreover, IL‐6 levels significantly correlated with PRc‐segment duration over time (*r* = .29, *p* = .020; Figure 5D), and this association was even more evident when COVID‐19 patients without any PR‐prolonging risk factor were selectively evaluated (*r* = .40, *p* = .030; Table 3). In these patients, also the strength of the correlation with CRP levels markedly increased up to reaching the statistical significance (PR‐segment: *r* = .42, *p* = .023; PRc‐interval: *r* = .41, *p* = .025; Table S6).

## 4 DISCUSSION

The key findings of the present study are the following: (1) During active disease, patients with severe COVID‐19 show a significant increase of ECG indices of AV conduction, specifically PR‐interval and PR‐segment; (2) the AV delay is transient as these parameters rapidly normalize, within days, during recovery; (3) PR‐indices significantly correlate with circulating IL‐6 levels over time; and (4) all these changes occur also in the absence of laboratory signs of cardiac strain/injury or concomitant treatment with PR‐prolonging drugs, repurposed or not.

In the recent years, accumulating data suggest that inflammatory activation may increase the AVB risk by negatively impacting cardiac conduction.^28^ In fact, besides promoting in the medium long‐term cardiac fibrosis^48^ (a well‐accepted mechanism for abnormal impulse propagation),^49^ several experimental and translational studies provided substantial evidence that inflammatory cytokines can rapidly induce gap‐junction dysfunction in cardiac myocytes. This by impairing expression, function, and distribution of connexins, particularly connexin‐40 and connexin‐43.^28^ Accordingly, it has been demonstrated that in patients with active inflammatory diseases, AV conduction is acutely delayed via an IL‐6‐mediated inhibition of connexin‐43 expression.^29^ Given that severe COVID‐19 is characterized by high‐grade systemic inflammation with elevated IL‐6 levels, it is likely that such mechanisms are also operative in this condition, possibly contributing to the higher risk of AVB observed.^36^, ^37^ However, while disease‐induced myocardial damage and cardiac toxicity of repurposed “off‐label” medications gained great attention as potential pathogenic factors,^10^, ^11^, ^12^, ^13^, ^14^, ^15^ at the moment there are no studies which have specifically evaluated the impact of IL‐6 elevation on COVID‐19‐associated conduction disturbances. To fill this gap, we here investigated changes in AV conduction indices and IL‐6 levels occurring in COVID‐19 patients during active phase and recovery, paying great attention to minimize the influence of concomitant confounding factors, particularly acute cardiac strain/injury and drug‐induced side effects.

In the present study, we found that in patients with severe COVID‐19, during the active phase of the disease, both PR‐interval and PRc‐interval were significantly prolonged when compared to healthy controls. Such alterations promptly reversed over days (median 7 days) after that therapeutic interventions, primarily glucocorticoids, led to disease recovery and systemic inflammation control. Specifically, by comparing active to recovery phase, mean PR/PRc‐intervals showed a ~ 11/15 ms prolongation, along with a 2.5‐times increase in the prevalence of I°AVB. Moreover, we also provided evidence that similar and even more evident changes were detectable when PRc‐segment was specifically evaluated, thereby suggesting that the conduction impairment observed in the active phase of the disease is mainly due to a transient depression of the AV node function. These data support and significantly expand the few information currently available on this subject. In fact, to date only two studies analyzed over time the acute impact of COVID‐19 on AV conduction, and although they consistently reported a significant delaying effect, both were based on retrospective data and focused on the PR‐interval only.^9^, ^18^ Our investigation confirmed the validity of these findings *for the first time* in a prospective sample of severe COVID‐19 patients, also when a more specific ECG parameter assessing AV nodal conduction was used (PR‐segment) and after applying Soliman‐Rautaharju's formula for correcting HR values (a parameter known to be markedly different in active vs. recovery phase of severe disease).

While the above new data per se are relevant, nevertheless the most important novel information deriving from our study is that such AV conduction abnormalities associate with the degree of systemic inflammatory activation, as reflected by circulating IL‐6 concentrations. This connection is robust as it persists independently from the presence of concomitant confounding factors potentially exerting PR‐prolonging effects. In fact, we found that in patients with severe COVID‐19 infection, there was a direct correlation over time between AV conduction times, particularly PRc‐interval and PRc‐segment, and circulating IL‐6 levels. Moreover, multiple sensitivity analyses provided evidence that this association remained, or even increased in strength (up to a *r* value of .40), also when patients with signs of acute myocardial involvement and/or treated with PR‐prolonging medications (COVID‐19 repurposed or classic) were excluded. Nonetheless, subjects with concomitant risk factors, particularly cardiac strain/injury, presented with mean PRc‐interval/segment values which have tendency to be longer when compared to those without. Such evidence, in connection with previous experimental studies demonstrating that conditions inducing stretch and injury of cardiac tissue can promote PR‐interval prolongation and other AV conduction disturbances,^50^, ^51^ points to synergistic deleterious effects of IL‐6 in the presence of other PR‐prolonging risk factors.

From a mechanistic point of view, the evidence here reported that the alterations in PR‐indices normalize in the span of a few days, a period of time not sufficient for structural heart changes (i.e., myocardial fibrosis), suggests that IL‐6 can delay AV conduction by directly and reversibly affecting cardiac electrophysiology. Accumulating recent data strongly support this hypothesis, in particular pointing to transient, but significant changes of connexins and other ion channels critically involved in the function of the AV node.^28^ In fact, IL‐6 potently down‐regulated connexin‐40 and connexin‐43 expression in cultured cardiomyocytes and macrophages,^29^, ^47^ an effect which was prevented by anti‐IL‐6 antibody preincubation.^47^ Accordingly, ex‐vivo studies in humans provided evidence that circulating IL‐6 levels associated indirectly with the expression of connexin‐40 and connexin‐43 in peripheral blood mononuclear cells (in turn strongly correlative of that measured in the myocardial tissue) and directly with ECG indices of AV conduction.^47^ Although IL‐6 can down‐regulate both connexins, connexin‐43 inhibition may have a primary role in explaining why the effects of this cytokine are so specific to AV node, due to the peculiar function that gap junctions containing connexin‐43 have in this part of the conduction system.^52^ In fact, it has been clearly demonstrated that in physiological conditions, cardiac macrophages are localized in the distal part of the AV node where crucially promote AV conduction by electrically coupling with conduction cardiomyocytes via gap junctions containing connexin‐43.^25^ Conditional deletion of connexin‐43 in macrophages or congenital absence of macrophages critically delays AV conduction,^25^ confirming the high physiological relevance of this mechanism. Given the central role played by this cell type in the immune‐inflammatory response, it is anticipated that IL‐6, representing one of the key cytokines involved in the regulation of the innate immune system,^53^ may have a particularly relevant impact on nodal cardiac macrophages.

In addition, a significant in‐vitro inhibitory activity on the L‐type calcium channel current I_CaL_ was reported in cardiac myocytes incubated with IL‐6.^14^ The in‐vivo relevance of these changes is supported by the evidence that acute intravenous or intraperitoneal administration of IL‐6 rapidly induced PR‐interval/PR‐segment prolongation in guinea pigs,^14^, ^29^ and tocilizumab, a monoclonal antibody against IL‐6 receptor, rescued such alterations.^14^ Notably, in the same animal and cell models, Zhu et al.^14^ also demonstrated that IL‐6 significantly enhanced both the AV conduction delaying potential (up to complete AV dissociation) and I_CaL_ inhibition induced by COVID‐19 repurposed drugs hydroxychloroquine and azithromycin. Given that, unlike the working myocardial cells, the upstroke phase of the action potential in AV nodal cells is mainly generated by I_CaL_,^54^ it is possible that these effects may also contribute to explain the specific impact of IL‐6 on AV conduction observed in our study.

Based on the above findings, it can be speculated that AV conduction abnormalities observed in our patients with severe COVID‐19 may be due, at least in part, to a transient IL‐6‐induced electric remodeling of the AV node leading to gap junction and/or L‐type calcium channel dysfunction.

Our work has several strengths and also some limitations. This is the first prospective study evaluating AV conduction in connection with IL‐6 levels in COVID‐19 patients, where, in addition to a reference sample of healthy controls, each subject represented her/his own control throughout the active and recovery phases of the disease. Moreover, in order to dissect the specific role of IL‐6 on the ECG parameters, significant effort was made to minimize the impact of concomitant confounding factors, by maintaining their presence stable over time and performing many sensitivity analyses. While such a study design strengthened the significance of our findings, however the relatively small sample size may represent a limitation warranting larger confirmatory studies.

In conclusion, our study *for the first time* provides evidence that in patients with severe COVID‐19 and high‐grade systemic inflammation, circulating IL‐6 elevation is associated with a significant delay of AV conduction, independent of concomitant confounding factors but synergistically operating with them. These changes promptly revert during the recovery phase in parallel with IL‐6 levels normalization, thereby suggesting that the underlying mechanism is a reversible, cytokine‐induced electrical remodeling of the AV node. While transient, such alterations may enhance the risk of adverse cardiac events, particularly severe AVB, a complication which occurs in a nonnegligible proportion of COVID‐19 patients increasing short‐term mortality.^6^, ^7^, ^8^, ^9^ In this regard, our data provide further support to current anti‐inflammatory treatment strategies employed in severe COVID‐19, including glucocorticoids and anti‐IL‐6‐targeted therapies (tocilizumab, sarilumab), as they could at the same time control respiratory involvement and reduce the risk of life‐threatening cardiac complications,^55^ specifically advanced AVB. In agreement with this hypothesis, a case of a 19‐year‐old COVID‐19 patient with multisystem inflammatory syndrome and new‐onset complete AVB improved to I°AVB after anti‐inflammatory treatment including methylprednisolone and tocilizumab has been recently reported.^56^ Moreover, evidence from meta‐analyses of randomized clinical trials indicates that glucocorticoids and/or IL‐6 antagonists can reduce short‐term cardiovascular death in patients with severe COVID‐19.^57^, ^58^ Further research on large samples of patients is warranted to assess the specific impact of these therapies on COVID‐19‐associated AVB and related outcomes.

## AUTHOR CONTRIBUTIONS

Conception and design of the work: P.E.L. Substantial contributions to the acquisition of data for the work: R.A., V.S., D.V., T.M., F.S., S.B., S.G., S.P., M.P., A.T., and A.O. Substantial contributions to the analysis of data for the work: P.E.L., R.A., A.C., and M.A. Substantial contributions to the interpretation of data for the work: P.E.L., R.A., M.A., F.L.P., M.A., M.B., and P.L.C. Drafting the work: P.E.L. and R.A. Revising the draft of the work critically for important intellectual content: M.A., A.C., F.L.P., L.C., P.L.C., and M.B. Final approval of the version to be published: R.A., P.E.L., V.S., A.C., D.V., T.M., F.S., S.B., G.C., S.G., S.P., M.P., A.T., A.O., F.L.P., M.A., M.B., and P.L.C. Agreement to be accountable for all aspects of the work in ensuring that questions related to the accuracy or integrity of any part of the work are appropriately investigated and resolved: R.A., P.E.L., V.S., A.C., D.V., T.M., F.S., S.B., G.C., S.G., S.P., M.P., A.T., A.O., F.L.P., M.A., M.B., and P.L.C.

## FUNDING INFORMATION

This work was supported by (1) Ministero dell'Istruzione, dell'Università e della Ricerca (MIUR), Progetti di Rilevante Interesse Nazionale (PRIN), Bando 2017, protocollo 2017XZMBYX (to Drs Lazzerini and Capecchi) and (2) Bando Ricerca COVID‐19 Toscana–2021, Progetto PRECARVID (to Drs Lazzerini and Capecchi).

## CONFLICT OF INTEREST STATEMENT

Dr. Pietro Enea Lazzerini received a grant from Roche Italia S.p.A. outside the submitted work, in 2018. The other authors declare no competing interests.

## ETHICS STATEMENT

Approval of the Research Protocol: Yes.

Informed Consent: Yes.

Registry and the Registration No. of the Study/Trial: N/A.

Animal Studies: N/A.

## Supporting information


Data S1:

## Data Availability

All data generated or analyzed during this study are included in this published article [and its supplementary information files].
