# Supplementary material for: Increased interleukin‐6 levels are associated with atrioventricular conduction delay in severe COVID‐19 patients
Source: J Arrhythm. 2024 Jul 31;40(5):1137–48. doi: 10.1002/joa3.13114 (PMC11474750; doi:10.1002/joa3.13114)
Supplement: Supplementary file 1 — Data S1: [file JOA3-40-1137-s001.docx]

# SUPPLEMENTAL INFORMATION

## Supplemental Methods

**Inter-observer and intra-observer variability in PR-interval and PR-segment measurement.** Although previous studies have documented acceptable intra-observer and inter-observer errors in measuring PR-interval and PR-segment,^1,2^ we evaluated intra-rater and inter-rater reliability within our center by estimating the intraclass correlation coefficient (ICC) and its 95% confidence interval based on the initial 20 measurements of PR-intervals and PR-segments conducted by two investigators (M.A. and R.A.). Specifically, for intra-rater reliability, we employed an ICC estimated on the model "two-way mixed effects, absolute agreement, single rater." Conversely, for inter-rater reliability, the ICC was estimated using the "two-way random effects, absolute agreement, single rater" model, following the guidelines provided by Koo and Li.^3^

The assessment of PR intervals and PR segments revealed robust repeatability and reproducibility within our study cohort. Intra-rater reliability analyses demonstrated a high level of consistency, with intraclass correlation coefficients (ICC) of 0.979 (95%CI: 0.947 to 0.991; p<0.001) for PR intervals and 0.957 (95%CI: 0.895 to 0.983; p<0.001) for PR segments. Similarly, inter-rater reliability analyses yielded ICC values of 0.979 (95%CI: 0.947 to 0.991; p<0.001) for PR intervals and 0.957 (95%CI: 0.895 to 0.983; p<0.001), indicative of an excellent level of reliability.

**References**

1. Yu YW, Huang Y, Zhao XM, et al. The prognostic predictive value of the components of the PR interval in hospitalized patients with heart failure. *BMC Cardiovasc Disord*. Mar 08 2023;23(1):119. doi:10.1186/s12872-022-03028-3

2. Lyhne MK, Debes KP, Helgogaard T, et al. Electrocardiography and heart rate variability in Göttingen Minipigs: Impact of diurnal variation, lead placement, repeatability and streptozotocin-induced diabetes. *J Pharmacol Toxicol Methods*. 2022;118:107221. doi:10.1016/j.vascn.2022.107221

3. Koo TK, Li MY. A Guideline of Selecting and Reporting Intraclass Correlation Coefficients for Reliability Research. *J Chiropr Med*. Jun 2016;15(2):155-63. doi:10.1016/j.jcm.2016.02.012

## Supplemental-Table I.

Demographic, laboratory and electrocardiographic characteristics of healthy controls.

Subjects, n 18

Age, years 63.5±11.5

Females, n 18 (33%)

CRP, mg/dl (r.v.<0.5) 0.2±0.3

IL-6, pg/ml (r.v.<7.1 pg/ml) 1.2±1.7

Heart rate, bpm 62.8±9.0

RR, ms 956.7±148.9

PR-interval,ms 152.0±18.4

PRc-interval, ms 149.5±18.6

PR-segment, ms 43.7±17.2

PRc-segment, ms 40.7±17.0

Subjects with PR-interval>99%CI*,n 4 (22%)

Subjects with PRc-interval PR>99%CI*, n 3 (17%)

Subjects with I° degree AVB**, n 0 (0%)

_______________________________________

CRP: C-reactive protein; IL-6: interleukin-6; PRc-interval: corrected PR-interval based on the Soliman’s formula; PRc-segment: corrected PR-segment based on the Soliman’s formula; RR: RR interval.

Values are expressed as mean±standard deviation or frequency count and percentages.

*PR-interval and PRc-interval >99% CI for age and sex in the general population

**PR-interval and/or PRc-interval >200 ms.

## Supplemental-Table II.

Changes in laboratory and electrocardiographic parameters in patients with COVID-19 (n=20) in absence of signs of cardiac strain/injury, during active disease and after therapeutic interventions resulting in an IL-6 decrease >60% when compared to the baseline.

ACTIVE RECOVERY *p*

CRP, mg/dl (r.v.<0.5) 10.2±6.2 1.0±1.2 **<0.001**

IL-6, pg/ml (r.v.<7.1 pg/ml) 30.7±20.3 3.2±2.0 **<0.001**

PR-interval,ms 158.8±20.3 148.1±22.1 **0.001**

PRc-interval, ms 159.8±22.1 147.0±22.4 **<0.001**

PR-segment, ms 48.2±14.8 41.3±13.4 **0.001**

PRc-segment, ms 49.3±16.5 39.5±13.8 **<0.001**

RR, ms 838.3±131.4 959.4±154.3  **0.003**

Heart rate, bpm 73.1±10.8 64.0 ±10.7 **0.005**

Troponin, ng/ml (r.v.<30) 8.0±6.5 7.3±5.4 0.554

BNP, pg/ml (r.v.<500) 147.8±123.2 172.4±156.0 0.602

paO_2_, mmHg (r.v.70-100) 93.2±47.6 101.0±36.7 0.220

paCO_2_ mmHg (r.v. 35-45) 35.2±3.3 35.0±4.6 0.949

pH (r.v.7.35-7.45) 7.46±0.0 7.45±0.0 0.286

P/F (r.v. >4.0) 2.2±1.1 2.9±0.9  **0.044**

_______________________________________

CRP: C-reactive protein; IL-6: interleukin-6; PRc-interval: corrected PR-interval based on the Soliman’s formula; PRc-segment: corrected PR-segment based on the Soliman’s formula; RR: RR interval; BNP: brain natriuretic peptide; P/F: paO_2_/FiO_2_ ratio; r.v.: reference values.

Values are expressed as mean±standard deviation.

Differences were evaluated by the two-tail Student’s paired “t” test, or the two-tail Wilcoxon matched pairs test.

## Supplemental-Table III.

Changes in laboratory and electrocardiographic parameters in patients with COVID-19 (n=26) in absence of repurposed COVID-19 drugs, during active disease and after therapeutic interventions resulting in an IL-6 decrease >60% when compared to the baseline.

ACTIVE RECOVERY *p*

CRP, mg/dl (r.v.<0.5) 9.9±8.8 0.7±0.9 **<0.001**

IL-6, pg/ml (r.v.<7.1 pg/ml) 32.5±22.3 3.8±2.7 **<0.001**

PR-interval,ms 168.1±29.5 158.6±30.0 **0.004**

PRc-interval, ms 170.2±29.4 156.6±28.8 **<0.001**

PR-segment, ms 57.9±27.8 48.8±23.8  **0.002**

PRc-segment, ms 60.0±27.7 46.8±22.6 **<0.001**

RR, ms 810.8±141.4 964.4±150.9  **0.003**

Heart rate, bpm 75.8±12.5 63.0±10.2 **0.002**

Troponin, ng/ml (r.v.<30) 18.3±33.8 13.2±12.6 0.997

BNP, pg/ml (r.v.<500) 425.6±476.5 277.5±302.4 0.361

paO_2_, mmHg (r.v.70-100) 88.3±40.5 99.3±24.3 0.110

paCO_2_ mmHg (r.v. 35-45) 35.4±4.0 36.1±4.4 0.600

pH (r.v.7.35-7.45) 7.46±0.0 7.45±0.0 0.449

P/F (r.v. >4.0) 2.1±1.0 2.7±0.9  **0.043**

_______________________________________

CRP: C-reactive protein; IL-6: interleukin-6; PRc-interval: corrected PR-interval based on the Soliman’s formula; PRc-segment: corrected PR-segment based on the Soliman’s formula; RR: RR interval; BNP: brain natriuretic peptide; P/F: paO_2_/FiO_2_ ratio; r.v.: reference values.

Values are expressed as mean±standard deviation.

Differences were evaluated by the two-tail Student’s paired “t” test, or the two-tail Wilcoxon matched pairs test.

## Supplemental-Table IV.

Changes in laboratory and electrocardiographic parameters in patients with COVID-19 (n=29) in absence of classic PR-prolonging drugs, during active disease and after therapeutic interventions resulting in an IL-6 decrease >60% when compared to the baseline.

ACTIVE RECOVERY *p*

CRP, mg/dl (r.v.<0.5) 9.8±8.3 0.8±1.0 **<0.001**

IL-6, pg/ml (r.v.<7.1 pg/ml) 38.9±42.7 3.6±2.7 **<0.001**

PR-interval,ms 165.1±27.6 152.8±25.5 **<0.001**

PRc-interval, ms 167.2±28.1 151.4±25.3 **<0.001**

PR-segment, ms 52.1±23.4 44.5±19.0 **<0.001**

PRc-segment, ms 54.2±23.8 43.1±18.8 **<0.001**

RR, ms 813.6±150.1 940.5±145.3 **<0.001**

Heart rate, bpm 75.9±13.7 64.7±10.4 **<0.001**

Troponin, ng/ml (r.v.<30) 15.7±31.8 11.0±12.0 0.550

BNP, pg/ml (r.v.<500) 426.1±499.2 260.0±290.8 0.255

paO_2_, mmHg (r.v.70-100) 90.8±42.4 101.8±33.2 0.057

paCO_2_ mmHg (r.v. 35-45) 35.2±3.3 35.9±4.8 0.552

pH (r.v.7.35-7.45) 7.46±0.0 7.45±0.0 0.372

P/F (r.v. >4.0) 2.2±1.4 2.7±1.0  **0.010**

_______________________________________

CRP: C-reactive protein; IL-6: interleukin-6; PRc-interval: corrected PR-interval based on the Soliman’s formula; PRc-segment: corrected PR-segment based on the Soliman’s formula; RR: RR interval; BNP: brain natriuretic peptide; P/F: paO_2_/FiO_2_ ratio; r.v.: reference values.

Values are expressed as mean±standard deviation.

Differences were evaluated by the two-tail Student’s paired “t” test, or the two-tail Wilcoxon matched pairs test.

## Supplemental-Table V.

Changes in laboratory and electrocardiographic parameters in patients with COVID-19 (n=15) in absence of cardiac strain/injury and repurposed COVID-19 drugs/classic PR-prolonging drugs, during active disease and after therapeutic interventions resulting in an IL-6 decrease >60% when compared to the baseline.

ACTIVE RECOVERY *p*

CRP, mg/dl (r.v.<0.5) 10.5±7.0 0.8±1.1 **<0.001**

IL-6, pg/ml (r.v.<7.1 pg/ml) 32.9±21.8 2.7±1.7 **<0.001**

PR-interval,ms 157.2±20.3 147.2±22.7 **0.006**

PRc-interval, ms 158.1±22.4 145.6±23.2 **0.002**

PR-segment, ms 49.2±15.2 41.2±13.5 **0.004**

PRc-segment, ms 50.1±16.9 39.6±13.8 **0.006**

RR, ms 842.2±131.6 958.0±164.9 0.092

Heart rate, bpm 72.3±9.9 64.5±11.5 **0.045**

Troponin, ng/ml (r.v.<30) 8.3±7.1 7.5±5.5 0.978

BNP, pg/ml (r.v.<500) 154.9±127.0 192.6±174.5 0.416

paO_2_, mmHg (r.v.70-100) 92.1±49.5 107.7±24.1 0.347

paCO_2_ mmHg (r.v. 35-45) 35.2±3.7 35.8±4.5 0.962

pH (r.v.7.35-7.45) 7.46±0.0 7.44±0.0 0.319

P/F (r.v. >4.0) 2.2±1.1 2.8±1.1 0.115

_______________________________________

CRP: C-reactive protein; IL-6: interleukin-6; PRc-interval: corrected PR-interval based on the Soliman’s formula; PRc-segment: corrected PR-segment based on the Soliman’s formula; RR: RR interval; BNP: brain natriuretic peptide; P/F: paO_2_/FiO_2_ ratio; r.v.: reference values.

Values are expressed as mean±standard deviation.

Differences were evaluated by the two-tail Student’s paired “t” test, or the two-tail Wilcoxon matched pairs test.

**Supplemental Table VI.**

Correlations between PR indices and C-reactive protein in the overall COVID-19 population

and in COVID-19 without specific or any PR-prolonging risk factor.

**PR-interval PRc-interval PR-segment PRc-segment**

**All patients** r=0.10 r=0.17 r=0.14 r=0.19

**(n=33)** p=0.41 p=0.18 p=0.26 p=0.12

**Patients without** r=0.07 r=0.15 r=0.16 r=0.20

**repurposed**  p=0.60 p=0.32 p=0.27 p=0.16

**COVID-19 drugs**

**(n=26)**

**Patients without** r=0.14 r=0.18 r=0.15 r=0.19

**classic PR-prolonging** p=0.31 p=0.15 p=0.15 0.14

**drugs**

**(n= 29)**

**Patients without** r=0.28 r=0.32 r=0.31 r=0.34

**cardiac strain/injury†** p=0.075 **p=0.042*** p=0.052  **p=0.042***

**(n=20)**

**Patients without** r=0.32 r=0.34 r=0.42 r=0.41

**any PR-prolonging** p=0.083 p=0.069  **p=0.023*** **p=0.025***

**risk factor**

**(n=15)**

_______________________________________

CRP: C-reactive protein; PRc-interval: corrected PR-interval based on the Soliman’s formula; PRc-segment: corrected PR-segment based on the Soliman’s formula; RR: RR interval; BNP: NT-pro-brain natriuretic peptide; P/F: paO_2_/FiO_2_ ratio; r.v.: reference values.

†Patients with both normal troponin (<30 ng/ml) and BNP (<500 pg/ml) levels.

Correlations were evaluated by the Spearman’s rank correlation. *p<0.05; **p<0.01.

Statistically significant p values (<0.05) are reported in bold.

**
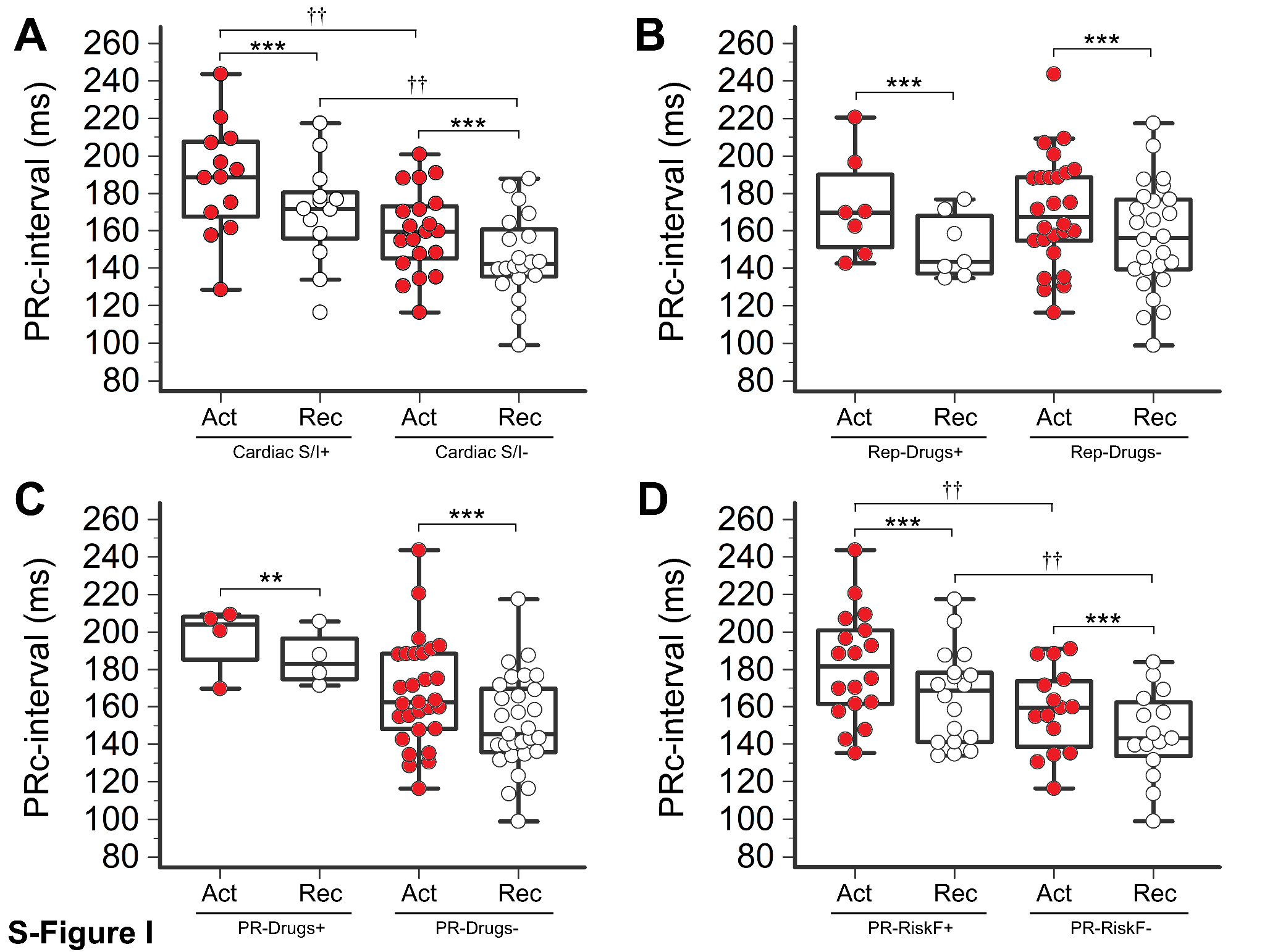
**


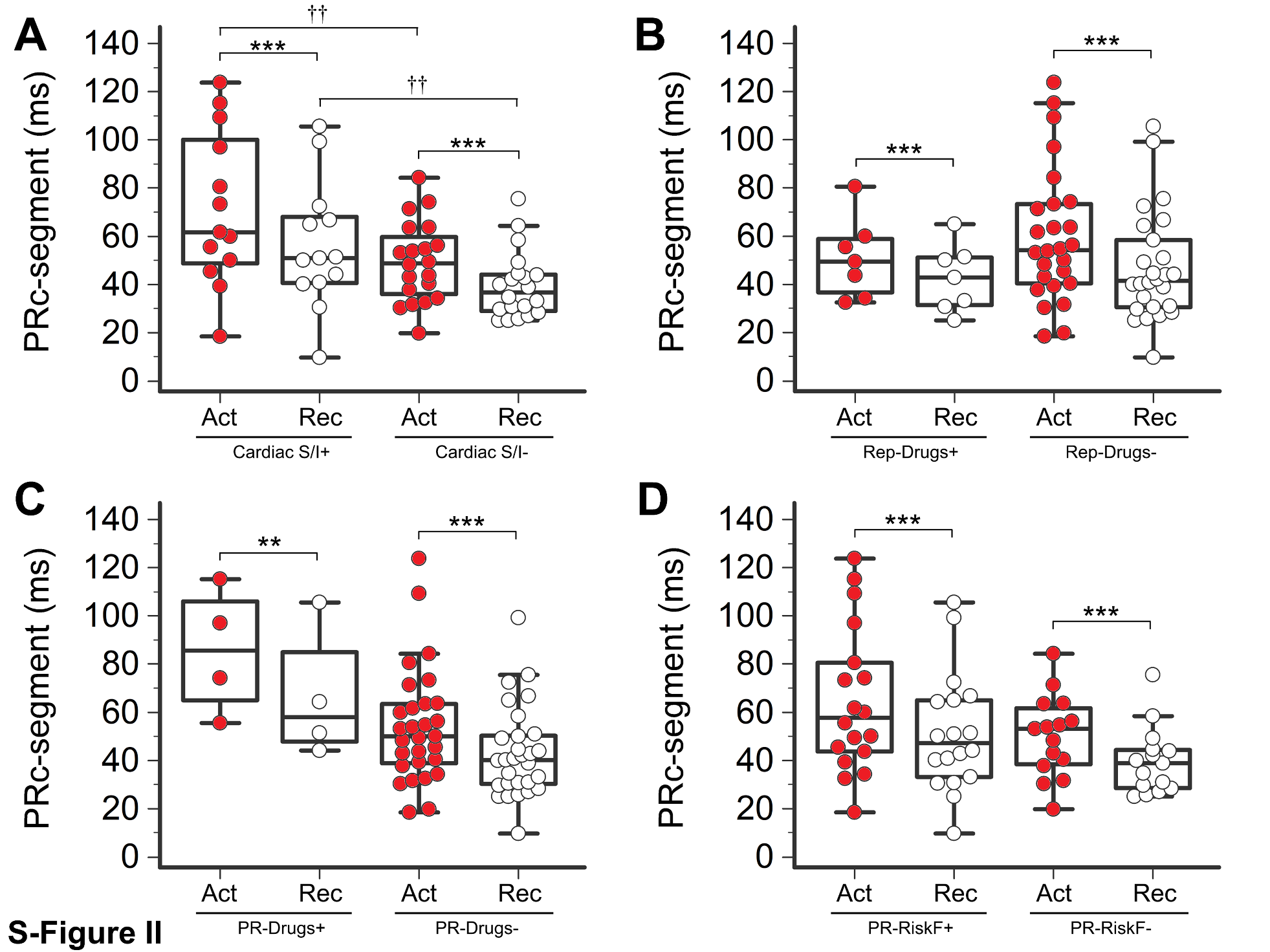


## LEGEND TO SUPPLEMENTAL FIGURES

**Supplemental-Figure I. PRc-interval in patients with COVID-19, during active disease (Act) and recovery (Rec), based on the presence or absence of concomitant PR-prolonging risk factors.** (**A**) PRc-interval in patients with (Cardiac S/I+, n=13) or without (Cardiac S/I-, n=20) cardiac strain/injury. (**B**) PRc-interval in patients with (Rep-Drugs+, n=7) or without (Rep-Drugs-, n=26) repurposed COVID-19 drugs. (**C**) PRc-interval in patients with (PR-Drugs+, n=4) or without (PR-Drugs-, n=29) classic PR-prolonging drugs. (**D**) PRc-interval in patients with (PR-RiskF+, n=18) or without (PR-RiskF-, n=15) any PR-prolonging risk factor. Two-tails paired t-test (***p<0.001, **p<0.01) or unpaired t-test (††p<0.01).

**Supplemental-Figure II. PRc-segment in patients with COVID-19, during active disease (Act) and recovery (Rec), based on the presence or absence of concomitant PR-prolonging risk factors.** (**A**) PRc-segment in patients with (Cardiac S/I+, n=13) or without (Cardiac S/I-, n=20) cardiac strain/injury. (**B**) PRc-segment in patients with (Rep-Drugs+, n=7) or without (Rep-Drugs-, n=26) repurposed COVID-19 drugs. (**C**) PRc-segment in patients with (PR-Drugs+, n=4) or without (PR-Drugs-, n=29) classic PR-prolonging drugs. (**D**) PRc-segment in patients with (PR-RiskF+, n=18) or without (PR-RiskF-, n=15) any PR-prolonging risk factor. Two-tails paired t-test (***p<0.001, **p<0.01) or unpaired t-test (††p<0.01).
